# Supplementary material for: Development and validation of COVID-19 vaccination perception (CoVaP) instrument among healthcare workers in Malaysia
Source: PeerJ. 2025 Apr 23;13:e19318. doi: 10.7717/peerj.19318 (PMC12032958; doi:10.7717/peerj.19318)
Supplement: Supplemental Information 3 [file peerj-13-19318-s003.docx]

Anda dikehendaki memilih hanya **SATU** jawapan berdasarkan pilihan jawapan yang disediakan. Tandakan (√) pada kotak yang berikut.

*You are required to choose only* ***ONE*** *answer based on the answer options provided. Tick ​​(√) in the following box.*

**Bahagian: Persepsi terhadap vaksin COVID-19.**

***Part: Perceptions towards COVID-19 vaccine.***

| **No.** | **Soalan *(Question)*** | **Sangat tidak setuju**  ***(Strongly disagree)*** | **Tidak setuju**  ***(Not agree)*** | **Neutral**  ***(Neutral)*** | **Setuju**  ***(Agree)*** | **Sangat setuju**  ***(Strongly agree)*** |
| --- | --- | --- | --- | --- | --- | --- |
| B2. | Saya berpendapat vaksin ini melindungi kita daripada komplikasi teruk jangkitan COVID-19.  *I think this vaccine protects us from the severe complications of COVID-19 infection.* |  |  |  |  |  |
| B3. | Saya berasa keberkesanan vaksin dapat mengatasi risiko kesan sampingan teruk vaksin.  *I feel the effectiveness of the vaccine outweighs the risk of serious side effects.* |  |  |  |  |  |
| B4. | Saya percaya vaksin COVID-19 selamat diambil kerana telah diuji secara klinikal.  *I believe the COVID-19 vaccine is safe because it has been clinically tested.* |  |  |  |  |  |
| B7. | Saya berpendapat vaksin mempunyai cip elektronik.  *I believe that this vaccine contains electronic chips.* |  |  |  |  |  |
| B8. | Saya percaya vaksin ini perlu diwajibkan kepada semua orang dewasa.  *I believe this vaccine should be mandatory for all adults.* |  |  |  |  |  |
| B9. | Saya meragui status halal vaksin COVID-19.  *I have a doubt about the COVID-19 vaccination halal status.* |  |  |  |  |  |
| B10. | Saya berpendapat perubatan alternatif (homeopati, herba dan lain-lain) boleh menggantikan vaksin bagi memberikan perlindungan daripada jangkitan COVID 19.  *I think alternative medicine (homeopathy, herbs, etc.) can replace vaccines to provide protection against COVID-19 infection.* |  |  |  |  |  |
